# Efficiency of lead aprons in blocking radiation − how protective are they?

**DOI:** 10.1016/j.heliyon.2016.e00117

**Published:** 2016-05-27

**Authors:** Seung-Jae Hyun, Ki-Jeong Kim, Tae-Ahn Jahng, Hyun-Jib Kim

**Affiliations:** Department of Neurosurgery, Spine Center, Seoul National University Bundang Hospital, Seoul National University College of Medicine, Seongnam, South Korea

**Keywords:** Medicine

## Abstract

**Background:**

Despite the firmly established occupational risk of exposure to X-rays, they are used extensively in spine surgeries. Shielding by lead aprons is the most common protective practice. We quantified the level of their radiation blocking ability in a real-life setting.

**Methods:**

Single-center, prospective, randomized study of adult patients with degenerative lumbar disorders, scheduled to undergo posterior lumbar interbody fusion. Instrumentation was performed in either a robot-assisted, minimally invasive approach (RO) or a conventional, fluoroscopically-assisted, open approach (FA). Outcome measures included the quantitative measurement of the surgeon’s actual exposure to radiation, as recorded by thermo-luminescent dosimeters (TLD) worn both above and under the 0.5 mm thyroid and trunk lead protectors.

**Findings:**

Sixty four patients were included in this study, 34 in the RO cohort and 30 in the FA cohort. The radiation blocked by the aprons, represented as the ratio of the under-apron to above-apron TLDs, averaged 37.1% (range 25.4–48.3%, 95% confidence interval between 30.6–43.6%). In the RO cohort, the average per-screw radiation dose and time were 51.9% and 73.7% lower, respectively, than the per screw exposure in the FA cohort.

**Interpretation:**

The 0.5 mm lead aprons blocked just over one third of the radiation scattered towards the surgeon. Use of robotic-guidance in a minimally invasive approach provided for a reduction of 62.5% of the overall radiation the surgeon was exposed to during open conventional approach. We conclude that reduced radiation use (e.g. by using robotic guidance) is a more effective strategy for minimizing exposure to radiation than reliance on protection by lead aprons, and recommend utilization of practices and technologies that reduce the surgical team’s routine exposure to X-rays.

## 1 Introduction

X-rays are used extensively in medical practice in general, and in orthopedic and spinal surgery in particular, due to its excellent imaging abilities of bony structures. Therefore, surgeons have come to depend on intraoperative X-ray-based imaging, mainly for anatomical orientation, instrumentation guidance and verification of execution, despite the fact that radiation exposure is a firmly established occupational hazard. The detrimental effects of radiation exposure have been thoroughly studied, both experimentally and epidemiologically [1], and can be classified as either stochastic or deterministic events, where the dose influences the probability and the magnitude of the event, respectively [2].

While mostly indirect, spine surgeons are chronically exposed to scatter beams, due to their proximity to the patient and radiation source. With the growing prevalence of minimally invasive approaches, radiation doses in the operating room have risen, predisposing the operating team to the deleterious effects of radiation. These include a range of ocular morbidities and tumors, thyroidal disorders, malignant solid neoplasms and leukemia. Mastrangelo et al. found an odds ratio of 5.4 for solid malignant tumors among orthopedic surgeons compared to healthcare workers in the same facility [3], but also noted that the surgeons reported poor compliance with radiation protection regulations.

The two main strategies for decreasing surgeon and operative staff exposure to radiation are: A) shielding by protective materials; and B) reducing exposure to X-rays, according to the ALARA (As Low As Reasonably Achievable) principle, by minimizing use of fluoroscopy, increasing the distance from the source, beam direction and collimation [4, 5, 6]. Shielding is mainly achieved by wearing protective lead aprons of 0.25 or 0.5 mm thickness, which have been cited to attenuate over 90% and 99% of the radiation dose, respectively [7]. Simon et al. demonstrated a radiation transmission range of 2.9–7.6% for 0.25 mm lead and 0.4–2.2% for 0.5 mm lead [8], concurring with previous studies [9, 10]. Other studies have reported radiation transmission factors ranging between 20–35% for 0.25 mm lead aprons [11, 12]. Yet, poor surgeon compliance with radiation safety guidelines is common, and seemingly stems from either a lack of awareness [3, 5], or from the ergonomic discomfort of wearing heavy lead aprons and their propensity to afflict surgeons with orthopedic disorders [10].

In this prospective study, we explored the empirical efficiency of protective lead aprons worn by the same surgeon performing spinal fusions in an open, fluoroscopically-assisted versus a minimally invasive, robotic-guided approach.

## 2 Materials and methods

### 2.1 Design

Single-center, prospective, randomized study of posterior lumbar interbody fusion performed in a robot-assisted, minimally invasive approach (RO) or a conventional, fluoroscopically-assisted, open approach (FA). Patients who signed the informed consent form were randomized (1:1) to undergo RO or FA spinal fusion. All the procedures were performed by the same spine surgeon. The study was registered on CRiS (WHO registration number: KCT0000993) and was approved by the local ethics committee (IRB No.: B-1311/228-008).

### 2.2 Patients

Adult patients presenting single or two-level degenerative lumbar spinal disorders scheduled to undergo primary fusion surgery, were eligible to participate in this study.

### 2.3 Surgical techniques

All patients underwent a spinal fusion by a posterior approach. In the RO cohort, a minimally invasive, para-spinal approach was used, where pilot holes for pedicle screw placement were drilled using robotic-assistance (Renaissance Surgical Guidance Robot, Mazor Robotics, Caesarea, Israel) as previously described [13]. Placement of interbody devices, and decompression when needed, were performed through the same paramedian incisions or in a mini-open midline incision using retractors and tubes. In the FA cohort, a midline incision was used to fully expose anatomical landmarks and pedicle screws were installed using fluoroscopic imaging for guidance and verification. Pedicle screws were percutaneously inserted by hand over guide-wires in the RO cohort. All procedures were performed using a C-arm fluoroscope (Siremobil; Siemens, Erlangen, Germany).

### 2.4 Outcome measures

**Fig. 1:**
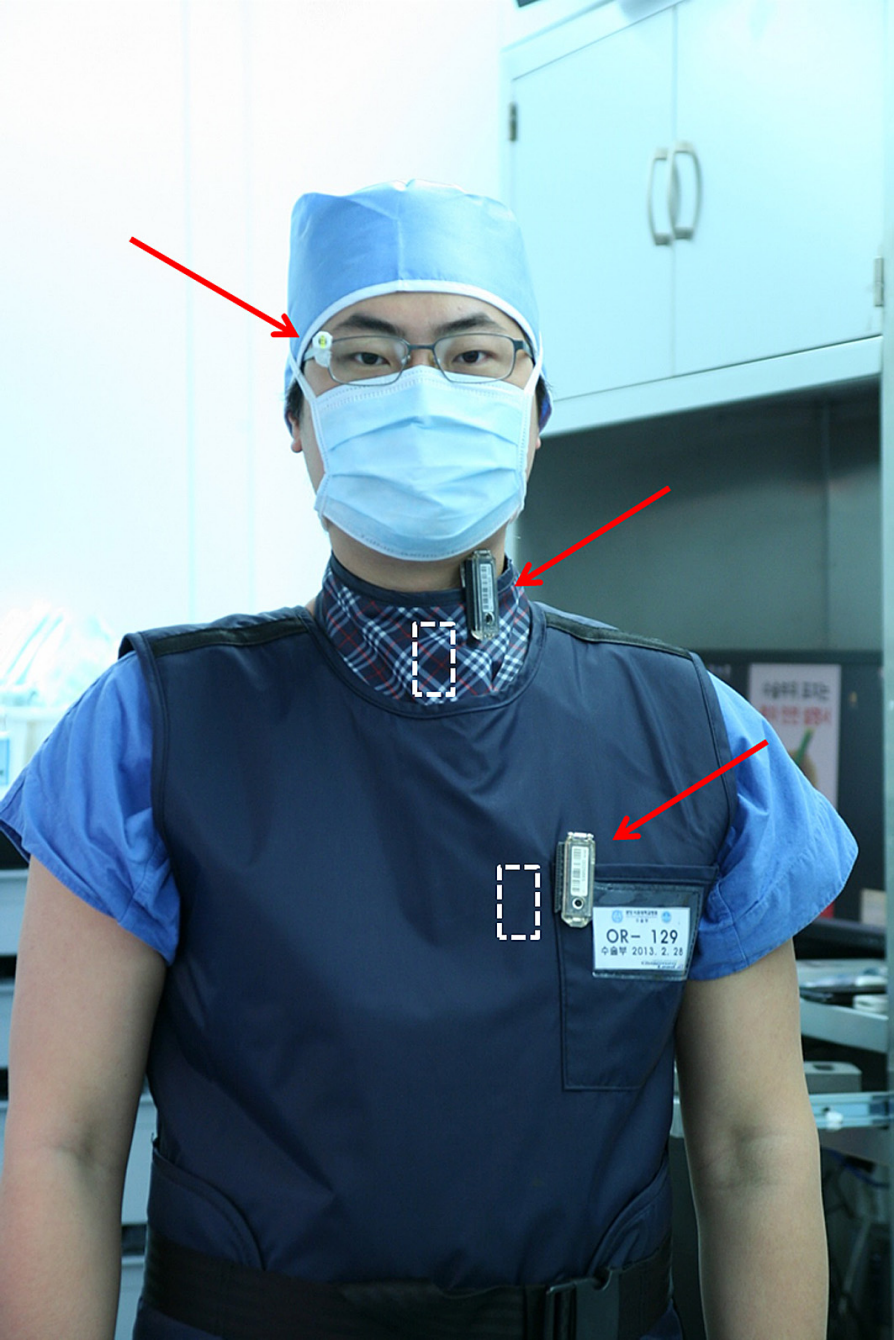
Placement of thermo-luminescent dosimeters.

Baseline data were collected, and included sex, age, height, weight and symptom duration. The clinical endpoints of the study will be reported elsewhere, once the data collection process is completed. In this report, we will focus on the quantitative measurement of intra-operative radiation exposure to the surgeon. These data include: 1) the direct operational output recorded by the C-arm in milli-Sieverts (mSv) and seconds of fluoro; 2) the surgeon’s exposure to radiation, recorded by thermo-luminescent dosimeters (TLD) (TLD-100, UD-802AT, Panasonic, Osaka, Japan). The TLDs were worn on: A) eye glasses; B) outside of thyroid protector; C) inside of thyroid protector; D) outside of trunk protector; E) inside of trunk protector (Fig. 1). Between the procedures, all dosimeters were stored in a radiation-free space and read by a blinded, independent institute (RADIN Co., Ltd, Daejeon, South Korea). The TLDs were read twice throughout the study; the RO TLDs were read after cases #17 and #34 and the FA TLDs were read after cases #12 and #30. The 0.5 mm-lead equivalent aprons (SK-15, Sung Kwang Meditech, Seoul, Korea) were arbitrarily selected from those available on hangers outside the operating room.

### 2.5 Statistical analysis

Difference in exposure to radiation between the RO and FA groups recorded by the TLDs was compared between the 2 groups as a ratio. Independent t-tests were used for the C-arm output data. All statistical analyses were performed using the SPSS 21.0.0 statistics package (SPSS, Inc., Chicago, IL). The level of statistical significance was set as alpha = 0.05.

## 3 Results

**Fig. 2:**
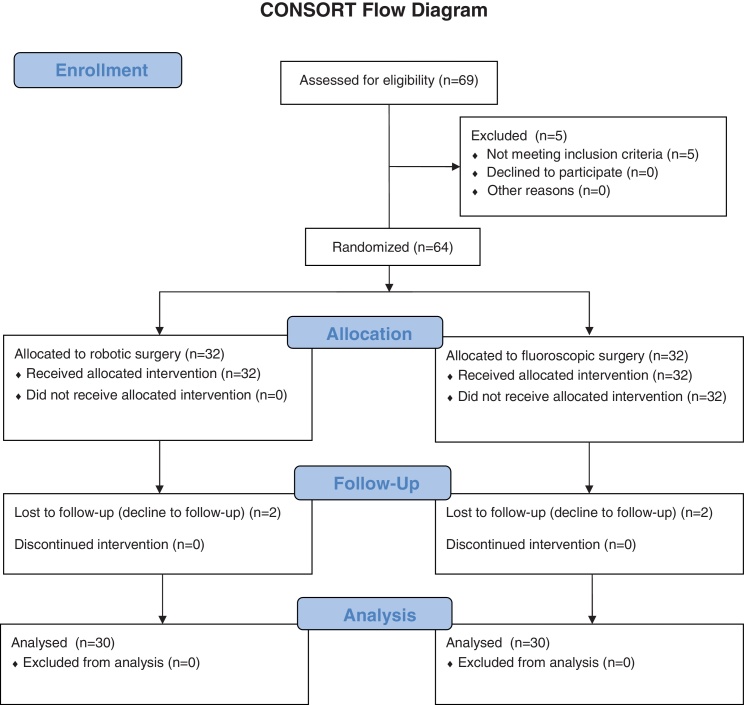
Profile of a Randomized Clinical Trial.

Sixty four patients were included in this study, 34 in the RO cohort and 30 in the FA cohort (Fig. 2). Four of the 34 RO cases were not included in the original randomized study but are included in this analysis, as the TLDs were worn by the surgeon during their surgery exactly as defined in the study protocol. Patients did not differ in age, gender, or indication for surgery (Table 1). A total of 154 screws were placed in the RO cohort (4.5 per patient), of which 140 were inserted in a percutaneous approach. There were 140 screws placed in the FA cohort (4.7 per patient), all inserted in an open approach.

The mean radiation dose emitted by the C-arm was 0.55 ± 0.40 in the RO cohort and 1.22 ± 1.14 mSv in the FA cohort (p < 0.001), with a mean 14.8 ± 9.0 and 59.7 ± 46.6 seconds of fluoroscopy (p < 0.001), respectively. The mean radiation dose per screw was 0.13 mSv and 0.27 mSv in the RO and FA cohorts, respectively (p < 0.001) and average fluoroscopy time per screw was 3.5 seconds and 13.3 seconds for the RO and FA cohorts, respectively (p < 0.001), amounting to a 51.9% reduction in fluoroscopy dose and a 73.7% reduction in fluoroscopy time, when using robotic guidance, as compared to the fluoroscopic assistance. Cumulative TLD absorption was consistently lower in the RO cohort, with a mean 62.5% (range 56.9–71.6%) reduction in overall radiation exposure per-screw during robot-guided surgeries, when compared to the FA procedures (Table 2).

The protection provided by the lead aprons was determined by the ratio of the under to above apron TLD measurements (Table 3). The mean amount of radiation blocked by the aprons was 37.1% (range 25.4–48.3%), with a 95% confidence interval of 30.6–43.6%. That is to say, that the transmission factor in our study ranged between 51.7% and 74.6%, representing the actual radiation dose absorbed by the surgeon.

## 4 Discussion

As modern spinal surgical practice becomes increasingly less invasive, the requisite instrumentation accuracy has come at the cost of heightened intraoperative radiation doses [14]. While the fields of interventional cardiology and radiology have seen the most extensive investigation of intraoperative exposure to X-ray [6, 10, 15], there is a growing body of evidence on safety aspects of fluoroscopically-guided procedures for spinal surgeons [16, 17, 18, 19]. However, these reports have often not found alarming doses according to the exposure guidelines [20, 21, 22].

This prospective study was designed to compare the chest, thyroid and eye occupational exposure to ionizing radiation during pedicle screw implantation procedures in either a robot-assisted, minimally invasive approach, versus a traditional, open approach, relying on 2D fluoroscopy for guidance and verification.

Fransen et al. found that the average radiation dose per pedicle screw was 3.2 times higher when inserted using a percutaneous versus open approach [23]. However, advances in image-guidance, namely navigation-systems coupled with intraoperative imaging systems (e.g. 2D- or 3D-fluoroscopy or intra-op CT), have enabled reduced use of intraoperative X-ray-based imaging and shorter overall surgery times. [24] In this study, we demonstrate that integration of robotic guidance was associated with reduced intraoperative use of radiation, as compared to the conventional freehand cohort (Tables 1, 2, and 3). The traditional open surgeries, which were performed in an open approach, used double or more radiation intraoperatively than in the minimally invasive, robotic-guided cohort. This finding is consistent with previous studies and mirrors Roser’s randomized study comparing MIS robotic-guidance with a traditional open freehand technique [25].

In this study we also assessed the effectiveness of lead aprons by thermoluminescent dosimeters positioned both above and beneath the protective lead aprons (Fig. 1, Table 3). In total, TLDs were read twice during the course of the study, providing four paired readings for determination of the protectiveness of the lead aprons in each treatment group. All paired readings consistently showed only partial protection of the apron, far below the expectations of surgeons wearing them, or the 99% radiation blocking cited by Bushberg et al. [7] Jackson et al. assessed different types of aprons by direct exposure to radiation and found dose reductions of up to 88% compared with no apron [26]. However, they assessed the effect of aprons directly in the beam path, rather than effects of scatter radiation, as surgeons encounter in the operating room. This was confirmed by Shousha et al. which demonstrated differences between different materials with a lead-equivalency of 0.5 mm, measuring transmission rates of 1–14% relative to beam strength [27]. Jones and Wagner found an even greater variance in performance of lead-equivalent materials relative to beam voltage (kVp) values, to the point of questioning the utility of measuring the lead equivalence of protective garments [28]. Christodoulou et al. used a more realistic approach as they simulated the environment to account for back scatter radiation. Their findings show similar variability in transmission factors, for different lead-equivalent aprons, with different beam strengths [9].

Harstall et al. prospectively assessed the radiation exposure directly to the surgeon during percutaneous vertebroplasties [20]. However they did not place TLDs directly under the protective apron, but rather a posterior “control” TLD on the surgeon’s exposed back, above the spine. When comparing the exposure over the thyroid protector to the back TLD, they found a decrease of about 95% of the radiation. Two potential explanations for this could be that their aprons were more effective [28, 29, 30] or that a portion of the radiation passing through the surgeon was absorbed in the vertebral column before hitting the posterior TLD. While they assessed percutaneous vertebroplasties, the per-pedicle radiation measured was equivalent to the doses measured in our study. The eye-level TLD was 0.017 mSv per pedicle, in both their study and ours in the FA cohort (in the RO cohort the exposure was 71.6% lower than the FA cohort, due to the use of robotic guidance). The radiation measured over the thyroid protector was 0.045 mSv in their study compared to 0.022 mSv in our FA cohort (0.008 in the RO cohort, Table 3). These results are within the range of per-case exposure levels found in other series assessing intraoperative use of fluoroscopy in a minimally invasive approach [20, 22, 26].

In our study, the TLD data did not correlate well with the emitted doses measured by the C-arms, especially in the robotic cohort (Table 1 versus Tables 2, 3). We believe that this is because the C-arm data are estimations for patient exposure based on beam output, while the TLD data represent the actual absorption of radiation scattered from the beam towards the surgeon. We believe that the differences in correlation in these parameters between the two study cohort are caused by the difference in the pattern of use of the C-arm. When working with the robot on screw placement, the Image Mode is used, which emits a higher dose per time unit. But the Standard Mode is used in the FA cohort, as well as in the robotic cohort during cage placement and decompression, resulting in a lower radiation dose per time unit. This difference in emission modes disrupts the linearity of dose vs. time and thus explains the poor correlation between mSv (as measured by the C-arm) and fluoroscopy seconds within the RO cohort, as well as between the two study cohorts.

The US National Council on Radiation Protection and Measurements (NCRP) limits the maximum annual total body dose for medical staff to 50 mSv, with a cumulative maximum lifetime limitation on exposure to radiation of 10 mSv per year of life of classified workers (e.g. radiologists), or 3 mSv for non-classified workers (e.g. spinal surgeons) [31]. The International Commission on Radiological Protection has stricter exposure limits, set at 20 mSv annually [32]. Both the NCRP and ICRP assume that the exposure to professionals is being done while they are exercising various protective measures. The maximum exposure allowed to the general public (i.e. unprotected individuals) from controllable sources is recommended by the Health Physics Society to not exceed 1 mSv annually above the annual natural background radiation (which is 3.1 mSv), with an effective dose up to 5 mSv per year in special (infrequent) circumstances [33]. At this dose, risks of radiation-induced health effects are either nonexistent or too small to be observed. The effects of cumulative lifetime exposures smaller than approximately 100 mSv in occupational workers, exposed to low levels of radiation, did not lead to radiation-related adverse health effects in the most reliable studies available [34]. When applying these thresholds to our results as measured under the apron in both trunk and thyroid, we calculated that they will be surpassed after about 1,600 surgeries in an open approach, the equivalent of about 16 years of work for a surgeon preforming 100 surgeries a year. If using robotic guidance in a minimally invasive approach we surpass the threshold of 100 mSv after 3,900 surgeries, the equivalent of about 39 years of work of performing 100 surgeries a year.

The main apparent weakness of this study is that we did not methodologically track the aprons being used, nor did we assess the effectiveness of the lead aprons by irradiating them directly in the beam path with TLDs above and under them. There were three considerations here: 1) we didn’t track the specific aprons used as we truly didn’t expect the results reported here; 2) we assessed the surgeon’s exposure to scatter radiation, which is a multidirectional effect. Assessing the direct blockage of radiation by the apron would reflect on the lead apron but would not be telling on the real life exposure of the surgeon; 3) the lead aprons worn during this study are used daily by operating room staff in a modern and well equipped, tertiary referral center and a leading teaching hospital in South Korea. We would assume that these results might occur at many, if not most, other facilities globally. It merits consideration and caution by surgeons who should reassess their protective practices, as these might not be as good as they believe, creating a false sense of safety.

This report does not address issues such as X-ray source position, collimation and other parameters that influence the absolute exposure, but rather, focuses on the protective capacities of the lead aprons. However, our absolute X-ray dosages are equivalent to those found in the professional literature in this type of surgeries.

Another potential limitation is the fact that only 8 pairs of TLD measurements were used in our study. However, each pair was used in 12–18 surgeries, averaging out the effect, and the results were quite consistent, with a 95% confidence interval of radiation blocked between 30.6–43.6%. However, it would be sound science if other centers would attempt to reproduce our results.

Mastrangelo et al. studied the incidence of cancer for orthopedic surgeons whose “radiation protection practice was poor”. Our results question whether use of lead aprons could be considered good protective practice?

In this prospective study assessing the extent lead aprons protect surgeons from intraoperative X-ray radiation emitted by C-arms, the 0.5 mm aprons used provided very partial protection, blocking only 37.1% of the radiation scattered towards the surgeon. Use of robotic-guidance in a minimally invasive approach demonstrated a reduction of 62.5% of the fluoroscopy dose compared to an open fluoroscopically-assisted approach, almost double the protection provided by the lead aprons in a freehand surgical technique. Therefore, we conclude that dose reduction is a more appropriate strategy than reliance on protection by lead aprons, and recommend utilization of practices and technologies that reduce the surgical team’s routine exposure to X-rays.

## Declarations

### Author contribution statement

Seung-Jae Hyun: Conceived and designed the experiments; Performed the experiments; Analyzed and interpreted the data; Contributed reagents, materials, analysis tools or data; Wrote the paper.

Ki-Jeong Kim, Tae-Ahn Jahng: Conceived and designed the experiments; Analyzed and interpreted the data; Contributed reagents, materials, analysis tools or data.

Hyun-Jib Kim: Analyzed and interpreted the data; Contributed reagents, materials, analysis tools or data.

### Funding statement

This research did not receive any specific grant from funding agencies in the public, commercial, or not-for-profit sectors.

### Competing interest statement

The authors declare no conflict of interest.

### Additional information

No additional information is available for this paper.

## Figures and Tables

**Table 1:** Baseline patient characteristics and surgical parameters by treatment cohort.

	RO	FA	p
Number of patients	34	30	
Age	66.5	66.8	0.916
Female (%)	70	73	
Body mass index	24.7	25.8	0.156
Number of levels fused	35	40	
Number of screws	154	140	
Operative time (skin to skin)	208.5	208.5	1.000
C-arm fluoro seconds (per screw)	3.5	13.3	0.000
C-arm mSv (per screw)	0.13	0.27	0.015

**Table 2:** Cumulative TLD absorption (mSv) by study arm and per screw.

TLD placement	Cumulative mSv		mSv per screw		% Reduction
	Robot-Guided (154 screws)	Fluoro-Guided (140 screws)	Robot-guided	Fluoro-guided	
Outside of thyroid protector	1.24	3.15	0.0082	0.0225	63.7%
Inside of thyroid protector	0.91	1.95	0.0060	0.0139	56.9%
Outside of trunk protector	1.37	3.35	0.0090	0.0239	62.3%
Inside of trunk protector	0.84	1.84	0.0055	0.0131	57.8%
Eye dosimeter	0.71	2.31	0.0047	0.0165	71.6%

**Table 3:** Readings of TLD pairs worn at the thyroid and trunk*.

Measurement	Outside Protector (mSv)	Inside Protector (mSv)	Protectiveness (i.e. radiation blocked)
Thyroid (RO1)	0.0083	0.0060	27.7%
Thyroid (RO2)	0.0080	0.0059	25.4%
Thyroid (FA1)	0.0222	0.0147	33.7%
Thyroid (FA2)	0.0226	0.0133	41.1%
Trunk (RO1)	0.0091	0.0056	38.0%
Trunk (RO2)	0.0089	0.0054	39.4%
Trunk (FA1)	0.0233	0.0121	48.3%
Trunk (FA2)	0.0243	0.0138	43.1%
*Average*			37.1 ± 7.7%

* TLDs were read twice during the course of the study. RO1–first readings of RO cohort TLDs, RO2- second reading of RO cohort TLDs, FA1–first reading of FA cohort TLDs, FA2–second reading of FA cohort TLDs.

## Appendix A Supplementary data

Study protocol
