# Supplementary material for: Efficiency of lead aprons in blocking radiation − how protective are they?
Source: Heliyon. 2016 May 27;2(5):e00117. doi: 10.1016/j.heliyon.2016.e00117 (PMC4946218; doi:10.1016/j.heliyon.2016.e00117)
Supplement: Study protocol [file mmc1.pdf]

STUDY PROTOCOL

This supplement contains the following items:
1. Original protocol, final protocol, but no changes.
2. Original statistical analysis plan, final statistical analysis plan, but no changes.

1. Background	
CRIS Registration Number	KCT0000993
Unique Protocol ID	B-1311/228-008
Public/Brief Title	Prospective randomized controlled trial of minimally invasive robotic- vs. fluoroscopic-assisted open pedicle screw placement in adult degenerative spinal fusion surgeries
Scientific Title	Prospective randomized controlled trial of minimally invasive robotic- vs. fluoroscopic-assisted open pedicle screw placement in adult degenerative spinal fusion surgeries
Acronym	Renaissance
MFDS Regulated Investigate	No
IND/IDE Protocol	No
Whether or not Registered at Other Registry	No

2. Institutional Review Board	
Board Approval Status	Submitted approval
Board Approval Number	B-1311/228-008
Approval Date	2013-12-12
Approval Submission	
Approval File	IRB_approval_report.zip
Board Name	Seoul National University Bundang Hospital Institutional Review Board
Data Monitoring Committee	No

3. Contact Details	
Contact Person for Principal Investigator/Scientific Queries	
- Name	Seung-Jae Hyun
- Degree	MD, PhD
- E-mail	sjhyun@snubh.cu.cc
- Tel	0317877164
- Institute	Seoul National University Bundang Hospital
Contact Person for Public Queries	
- Name	Seung-Jae Hyun
- Degree	MD, PhD
- E-mail	sjhyun@snubh.cu.cc
- Tel	0317877164
- Institute	Seoul National University Bundang Hospital
Contact Person for Updating Information	

– Name	Seung-Jae Hyun
– Degree	MD, PhD
– E-mail	sjhyun@snubh.cu.cc
– Tel	0317877164
– Institute	Seoul National University Bundang Hospital

4. Status	
Study Organization	Single
Overall Recruitment Status	Recruiting
Date of First Enrollment	2013-12-13
Type of Enrollment	Actual
Target Sample Size	60
Primary Completion Date	2014-12-11
Study Completion Date	2015-12-11
Participation Recruitment Status1	
– Institute Name	Seoul National University Bundang Hospital
– Recruitment Status	Recruiting
– Date of First Enrollment	2013-12-13
– Type of Enrollemnt	Actual

5. Source of Monetary/Material Support	
Source of Monetary/Material Support 1	
– Organization Name	Seoul National University Bundang Hospital
– Organization Type	Medical Institute
– Project ID	B-1311-228-008
**6. Sponsor Organization**	
Sponsor Organization 1	
– Institute Name	Seoul National University Bundang Hospital
– Institute Type	Medical Institute
**7. Study Summary**	
Lay Summary	To quantify potential short- and long-term benefits of robotically-guided minimally invasive spine surgery (MIS) in instrumentation of degenerative thoracic, lumbar or lumbosacral spine disease in adult patients, in comparison to instrumentation in a matching cohort of control patients performed using conventional fluoroscopic-assisted technique.<br><br>It is hypothesized that use of robotic guidance during spinal instrumentation will have numerous short- and long-term benefits to both patient and surgeon, relative to image-guided MIS techniques. Expected benefits include improved implant accuracy, lower intraoperative or perioperative complications including reduced operation time and blood loss, lower incidence of procedure-related adverse events (e.g., new neurological deficit, implant-related durotomies, implant misplacement), lower reoperation rates, lower incidence of implant failure (e.g., screw

pullout), improved surgical outcomes, a higher ratio of executed:planned instruments, fewer "abandoned" screws.

Primary Endpoints
- Pedicle screw instrumentation accuracy scored using the Gertzbein-Robbins classification of postoperative CTs in a subset of patients
- Outcome measures included the quantitative measurement of the surgeon's actual exposure to radiation as recorded by thermo-luminescent dosimeters (TLD) worn both above and under the thyroid and trunk protectors.
- Clinical outcome measures assessed using questionnaires (e.g., back and leg VAS, ODI, SF-36)
- Operation duration, intraoperative blood loss, postoperative drainage, total blood loss, amount of transfusion, time to ambulation
- Surgical complications (e.g., new neural deficits, implant-related durotomy, infection requiring surgery, excessive blood loss [> 40% of blood volume])
- Revision surgeries, reoperation including incidence of adjacent segment degeneration

Robot group : Patients will be selected to participate in the study according to the inclusion/exclusion criteria listed below. Patients will be randomly selected in the robotic surgery arm.
Fluoroscope group : Randomly selected patients will participate in the control arm only. Surgeons comfortable enough with both surgical techniques will randomize their first 60 patients (1:1 ratio, 30 patients to each study arm).
In either arm, patients will undergo the surgical intervention according to standard hospital care and surgical techniques - with or without robotic guidance, according to the study arm.

Inclusion Criteria
1. Adult patients (age from 21 years), undergoing thoracic or lumbar or lumbosacral spinal fixation surgery.
2. Include spinopelvic fusion surgeries involving iliac screws, although these screws will not be included in the data analysis.
3. Primary surgery only
4. Patient capable of complying with study requirements
5. Signed informed consent of patient or legal guardian

Exclusion Criteria
1. Infection or malignancy
2. Primary abnormalities of bones (e.g. osteogenesis imperfecta, congenital or idiopathic spinal deformities)
3. Primary muscle diseases, such as muscular dystrophy
4. Neurologic diseases (e.g. Charcot-Marie Tooth, Guillain-Barre syndrome, cerebral palsy,

	spina bifida, or neurofibroma)<br>5. Spinal cord abnormalities with any neurologic symptoms or signs<br>6. Spinal cord lesions requiring neurosurgical interventions, such as hydromyelia<br>7. Paraplegia<br>8. Patients requiring anterior release or instrumentation<br>9. Any other significant disease or disorder which, in the opinion of the Investigator, may either put the participants at risk because of participation in the study, or may influence the result of the study.<br>10. Patient cannot follow study protocol, for any reason<br>11. Patient (or legal guardian, when applicable) cannot or will not sign informed consent

8. Study Design		
Study Type		Interventional Study
Primary Purpose		Treatment
Phase		Phase4
Intervention Model		Parallel
Blinding/Masking		Single
Blinded Subject		Subject, Caregiver
Allocation		RCT
Intervention Type		Device, /Procedure/Surgery
Intervention Description		Robot group : Patients will be selected to participate in the study according to the inclusion/exclusion criteria listed below. Patients will be randomly selected in the robotic surgery arm.<br>Fluoroscope group : Randomly selected patients will participate in the control arm only. Surgeons comfortable enough with both surgical techniques will randomize their first 60 patients (1:1 ratio, 30 patients to each study arm). In either arm, patients will undergo the surgical intervention according to standard hospital care and surgical techniques – with or without robotic guidance, according to the study arm.
Number of Arms		2
Arm 1	Arm Label	Robot group
	Target Sample Size	30
	Arm Type	experimental
	Arm Description	Robot group : Patients will be selected to participate in the study according to the inclusion/exclusion criteria listed below. Patients will be randomly selected in the robotic surgery arm.
Arm 2	Arm Label	Fluoroscope group
	Target Sample Size	30

	Arm Type	active comparator
	Arm Description	Fluoroscope group : Randomly selected patients will participate in the control arm only. Surgeons comfortable enough with both surgical techniques will randomize their first 60 patients (1:1 ratio, 30 patients to each study arm).

9. Subject Eligibility		
Condition(s)/Problem(s)		* Diseases of the musculo-skeletal system and connective tissue
Rare Disease		No
Inclusion Criteria	Gender	Both
	Age	21 Year ~ N/ANo Limit
	Description	1. Adult patients (age from 21 years), undergoing thoracic or lumbar or lumbosacral spinal fixation surgery.<br>2. Include spinopelvic fusion surgeries involving iliac screws, although these screws will not be included in the data analysis.<br>3. Primary surgery only<br>4. Patient capable of complying with study requirements<br>5. Signed informed consent of patient or legal guardian
Exclusion Criteria		1. Infection or malignancy<br>2. Primary abnormalities of bones (e.g. osteogenesis imperfecta, congenital or idiopathic spinal deformities)<br>3. Primary muscle diseases, such as muscular dystrophy<br>4. Neurologic diseases (e.g. Charcot-Marie Tooth, Guillain-Barre syndrome, cerebral palsy, spina bifida, or neurofibroma)<br>5. Spinal cord abnormalities with any neurologic symptoms or signs<br>6. Spinal cord lesions requiring neurosurgical interventions, such as hydromyelia<br>7. Paraplegia<br>8. Patients requiring anterior release or instrumentation<br>9. Any other significant disease or disorder which, in the opinion of the Investigator, may either put the participants at risk because of participation in the study, or may influence the result of the study.<br>10. Patient cannot follow study protocol, for any reason<br>11. Patient (or legal guardian, when applicable) cannot or will not sign informed consent
Statistical considerations		Statistical analysis<br>Descriptive statistics of demographic data will be presented for the entire study population.<br><br>Statistics will be provided also for all the primary and secondary endpoints, including at a minimum mean and standard deviation, and compared between robotic surgery and control arms. Strong trends that are identified in the data will also be analyzed for statistical significance and power.<br><br>Data analysis<br>The data will be aggregated into two groups (robotic surgery and fluoroscopic control arms) by the principal investigator or a designee, and de-identified as to patient information as well as surgical technique (with/without robotic guidance) information. The de-identified data will be analyzed.<br><br>Descriptive statistics will be provided for all demographic data and relevant endpoints. The means and standard deviations (SD) of quantitative variables will be calculated and compared between the two arms. Paired t-tests or signed rank tests for two rank tests for paired observations will be applied to determine the statistical significance of the changes measured.<br><br>Alpha is defined as 5%.

10. Outcome Measure(s)	
Type of Primary Outcome	/Safety/Efficacy
Primary Outcome 1	
- Outcome	Pedicle screw instrumentation accuracy scored using the Gertzbein-Robbins classification of postoperative CTs in a subset of patients
- Timepoint	6-12 months after surgery
Primary Outcome 2	
- Outcome	Clinical outcome
- Timepoint	until 2 years after surgery
Primary Outcome 3	
- Outcome	Complications
- Timepoint	until 2 years after surgery
Primary Outcome 4	
- Outcome	Radiation dose/time
- Timepoint	Operation day
Secondary Outcome 1	
- Outcome	Clinical performance of instrumentation technique
- Timepoint	Operation day
Secondary Outcome 2	
- Outcome	Hospital days
- Timepoint	after discharge

11. Publication		
Publication	0	

<https://cris.nih.go.kr/cris/search/search_result_st01_en.jsp?seq=4637&type=my>
